# Newly Developed Fully Covered Metal Stent for Unresectable Malignant Biliary Stricture

**DOI:** 10.1155/2010/903520

**Published:** 2010-10-19

**Authors:** Kei Ito, Naotaka Fujita, Yutaka Noda, Go Kobayashi, Takashi Obana, Jun Horaguchi, Shinsuke Koshita, Yoshihide Kanno, Takahisa Ogawa, Yuhei Kato, Yasunobu Yamashita

**Affiliations:** Department of Gastroenterology, Sendai City Medical Center, 5-22-1, Tsurugaya, Miyagino-ku, Sendai, Miyagi 983-0824, Japan

## Abstract

We herein report two patients with unresectable malignant biliary stricture who underwent stenting with a newly developed fully-covered metal stent. In the first case of lower-middle bile duct cancer, a stent was placed through the stenosis. In the second case of middle bile duct stricture due to lymph node metastases from gallbladder cancer, a stent was placed in the bile duct across the stenosis. No procedure-related complications were observed. Unevenness of the outer surface and a low shortening ratio are expected to lessen the occurrence of complications characteristic of covered metal stents such as stent migration and bile duct kinking.

## 1. Introduction

Endoscopic biliary stenting is an efficacious treatment for patients with unresectable malignant biliary stricture. In spite of the wider caliber of a self-expandable metal stent (SEMS), which has a longer stent patency than that of plastic stents [1–5], occlusion by tumor/tissue ingrowth can develop due to the meshwork design. Although covered metal stents have been reported to contribute to prevention of tumor/tissue ingrowth [6], issues of liability to particular complications such as stent migration and kinking of the bile duct have not been resolved [7, 8]. We herein report two patients with unresectable malignant biliary stricture who successfully underwent placement of a newly developed fully-covered metal stent, characterized by an uneven outer surface, a low shortening ratio, and a low axial force.

## 2. Case 1

 An 80-year-old man was admitted to our department, presenting with jaundice and a high fever. He had undergone cholecystectomy due to cholecystolithiasis. Laboratory data showed elevation of hepatobiliary enzymes and C-reactive protein. Abdominal enhanced computed tomography (CT) revealed wall thickening of the lower-middle bile duct with upstream dilation of the upper bile duct. MR cholangiopancreatography revealed a strictures of the lower-middle bile duct and a normal pancreatic duct.

**Figure 1:**
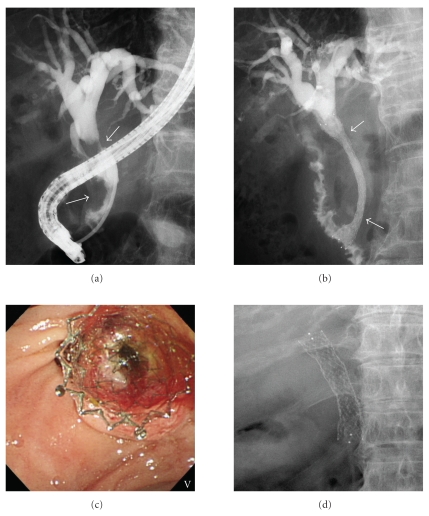
(a, b) Endoscopic retrograde cholangiography; (c) duodenoscopy; (d) fluoroscopy. ERC revealed strictures (arrows), 4 cm in length, in the lower-middle bile duct (a). A fully-covered metal stent (arrows) (covered Zeostent), 8 cm in length and 1 cm in diameter, was placed through the papilla of Vater and the stricture (b, c). The stent was fully expanded the day after the procedure (d).

**Figure 2:**
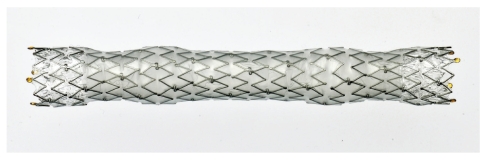
Covered Zeostent.

 Endoscopic retrograde cholangiography (ERC) revealed a strictures, 5 cm in length, in the lower-middle bile duct (Figure 1(a)). Transpapillary intraductal ultrasonography of the bile duct revealed a tumor, spreading from the lower bile duct to the upper bile duct. Based on the diagnosis of widespread bile duct cancer, an 8 Fr plastic stent was placed through the biliary stricture up to the upper bile duct for biliary decompression following sphincterotomy. Ten days after the procedure, replacement of the plastic stent with a covered metal stent was attempted as a palliative therapy after obtaining informed consent. Using a duodenoscope (TJF-260 V: Olympus Medical Systems, Co., Ltd. Tokyo, Japan), a fully-covered metal stent (covered Zeostent: Zeon Medical Inc., Tokyo, Japan) (Figure 2), 8 cm in length and 1 cm in diameter, was placed through the stricture (Figures 1(b) and 1(c)). There were no clinical symptoms suggesting development of pancreatitis. The placed stent was fully expanded the day after the procedure (Figure 1(d)). After improvement of jaundice, the patient received chemotherapy. No complications such as stent migration and occlusion were observed during the five-month followup period.

## 3. Case 2

 A 77-year-old man presenting with right upper quadrant pain was referred to our department for further evaluation of a tumor mass in the gallbladder revealed by ultrasonography. Laboratory data showed elevation of hepatobiliary enzymes. Abdominal enhanced CT showed the tumor to be in the fundus of the gallbladder, directly invading the hepatic parenchyma, and multiple swollen lymph nodes along the extrahepatic bile duct.

**Figure 3:**
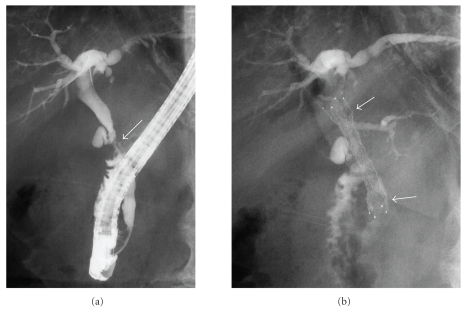
Endoscopic retrograde cholangiography. ERC revealed a stricture (arrow), 1.5 cm in length, in the middle bile duct (a). A covered Zeostent (arrow), 6 cm in length and 1 cm in diameter, was placed in the bile duct across the stricture following sphincterotomy (b).

 Based on the diagnosis of gallbladder cancer with lymph node metastases, transpapillary biliary stenting was attempted after obtaining written informed consent. ERC revealed a stricture, 1.5 cm in length, in the middle bile duct (Figure 3(a)). A covered Zeostent, 6 cm in length and 1 cm in diameter, was inserted into the bile duct across the stricture and the cystic duct up to the upper bile duct following sphincterotomy (Figure 3(b)). There were no clinical symptoms suggesting complications such as pancreatitis and cholecystitis. The placed stent was fully expanded the day after the procedure. Stent migration and other complications were not observed during the four-month followup period.

## 4. Discussion

 SEMS placement is a widely accepted treatment for unresectable malignant biliary stricture. Nowadays, several types of SEMS are commercially available. Membrane-coated SEMSs were developed to prevent tumor/tissue ingrowth through the metal latticework. Isayama et al. [6] reported a randomized controlled trial (RCT), enrolling 112 patients with unresectable malignant biliary stricture for endoscopic insertion of either a covered Diamond stent (*n* = 57) or a noncovered Diamond stent (*n* = 55). They reported a higher cumulative patency rate in the covered Diamond stent group, whereas more recent retrospective studies, comparing a covered Wallstent with a noncovered Wallstent, did not demonstrate improved stent patency in the covered stent group [8, 9]. This discrepancy may be explained by the difference of density of meshwork between a Diamond stent and a Wallstent.

 At present, neither a covered Diamond stent nor a covered Wallstent is commercially available. As for covered SEMS, WallFlex Biliary RX Stent (Microvasive, Boston Scientific Corp., Natick, MA, USA), ComVi stent (Taewoong Medical Inc., Seoul, Korea), and GORE VIABIL biliary endoprosthesis (W. L. Gore & Associates, Inc., Newark, DE, USA) are commercially available in Japan. Covered Zeostent is a newly developed fully-covered SEMS.

 Covered SEMSs entail liability to particular complications such as stent migration, pancreatitis, and cholecystitis. Characteristics of SEMS are determined by various factors such as material, structure, shortening ratio, and radial/axial force [10]. A high axial force or high shortening ratio is thought to cause kinking of the bile duct or stent migration, respectively, after deployment of the stent. There are several characteristics unique to covered Zeostent other than its fully covered structure. One is the shape of the stent after full expansion. It has a wavy contour with an uneven outer surface, which expectedly contributes to prevention of stent migration. A very low shortening ratio (about 3%) is another characteristic, which facilitates accurate deployment of the stent. Furthermore, the axial force is lower compared with that of other covered SEMSs [10], which expectedly avoids kinking of the bile duct.

 There may be some drawbacks with this particular stent. When a stent is placed through the papilla of Vater as in Case 1, post-ERCP pancreatitis may develop due to occlusion of the pancreatic duct orifice. Cholecystitis also should be taken into consideration, as is the case with other covered SEMSs. Although no procedure-related complications were observed in either patient in a short-term followup, accumulation of patients is necessary for elucidation of the utility of this stent.

 In conclusion, placement of this newly developed fully-covered metal stent (covered Zeostent) for unresectable malignant biliary stricture appears to be safe and efficacious. Data on long-term outcome and adequate RCTs to enable comparison with other covered SEMS are awaited for assessment of the safety and effectiveness of this stent.
